# Plant innate immunity against human bacterial pathogens

**DOI:** 10.3389/fmicb.2014.00411

**Published:** 2014-08-11

**Authors:** Maeli Melotto, Shweta Panchal, Debanjana Roy

**Affiliations:** ^1^Department of Plant Sciences, University of CaliforniaDavis, CA, USA; ^2^Department of Biology, University of TexasArlington, TX, USA

**Keywords:** leafy vegetables, fresh produce, *Salmonella enterica*, *Escherichia coli* O157:H7, plant defense

## Abstract

Certain human bacterial pathogens such as the enterohemorrhagic *Escherichia coli* and *Salmonella enterica* are not proven to be plant pathogens yet. Nonetheless, under certain conditions they can survive on, penetrate into, and colonize internal plant tissues causing serious food borne disease outbreaks. In this review, we highlight current understanding on the molecular mechanisms of plant responses against human bacterial pathogens and discuss salient common and contrasting themes of plant interactions with phytopathogens or human pathogens.

## Introduction

Bagged greens in the market are often labeled “pre-washed,” “triple-washed,” or “ready-to-eat,” and look shiny and clean. But are they really “clean” of harmful microbes? We cannot be so sure. Food safety has been threatened by contamination with human pathogens including bacteria, viruses, and parasites. Between 2000 and 2008, norovirus and *Salmonella* spp. contributed to 58 and 11% of forborne illnesses, respectively in the United States (Scallan et al., 2011). In those same years, non-typhoidal *Salmonella* alone was ranked as the topmost bacterial pathogen contributing to hospitalizations (35%) and deaths (28%) (Scallan et al., 2011). In 2007, 235 outbreaks were associated with a single food commodity; out of which 17% was associated with poultry, 16% with beef, and 14% with leafy vegetables that also accounted for the most episodes of illnesses (CDC, 2010).

Apart from the direct effects on human health, enormous economic losses are incurred by contaminated food products recalls. The 8-day recall of spinach in 2006 cost $350 million to the US economy (Hussain and Dawson, 2013). It should be realized that this is not the loss of one individual, but several growers, workers, and distributors. This is a common scenario for any multistate foodborne outbreak. Additionally, the skepticism of the general public toward consumption of a particular food product can lead to deficiencies of an important food source from the diet. Less demand would in turn lead to losses for the food industry. Economic analysis shows that money spent on prevention of foodborne outbreak by producers is much less than the cost incurred after the outbreak (Ribera et al., 2012).

Contamination of plants can occur at any step of food chain while the food travels from farm to table. Both pre-harvest and post-harvest steps are prone to contamination. Contaminated irrigation water, farm workers with limited means of proper sanitation, and fecal contamination in the farm by animals can expose plants to human pathogens before harvest of the edible parts (Lynch et al., 2009; Barak and Schroeder, 2012). After harvest, contamination can occur during unclean modes of transportation, processing, and bagging (Lynch et al., 2009). Mechanical damage during transport can dramatically increase the population of human pathogens surviving on the surface of edible plants (Aruscavage et al., 2008). Control measures to decrease pathogen load on plant surfaces have been defined by the Food Safety Modernization Act (US Food and Drug Administration) and Hazard Analysis and Critical Control Point system (HACCP). Using chlorine for post-harvest crop handling has been approved by US Department of Agriculture (USDA) under the National Organic Program. However, some studies indicated that internalized human pathogens escape sanitization (Seo and Frank, 1999; Saldaña et al., 2011). Thus, understanding the biology of human pathogen-plant interactions is now crucial to prevent pathogen colonization of and survival in/on plants, and to incorporate additional, complementing measures to control food borne outbreaks.

We reasoned that as plants are recognized vectors for human pathogens, enhancing the plant immune system against them creates a unique opportunity to disrupt the pathogen cycle. In this cross-kingdom interaction, the physiology of both partners contribute to the outcome of the interactions (i.e., colonization of plants or not). Bacterial factors important for interaction with plants have been discussed in recent, comprehensive reviews (Tyler and Triplett, 2008; Teplitski et al., 2009; Berger et al., 2010; Barak and Schroeder, 2012; Brandl et al., 2013). Plant factors contributing to bacterial contamination (or lack of) is much less studied and discussed. In this review, we highlight current knowledge on plants as vectors for human pathogens, the molecular mechanisms of plant responses to human bacterial pathogens, and discuss common themes of plant defenses induced by phytopathogens and human pathogens. We have focused on human bacterial pathogens that are not recognized plant pathogens such as *Salmonella enterica* and *Escherichia coli* (Barak and Schroeder, 2012; Meng et al., 2013), but yet are major threats to food safety and human health.

## Plant surface: the first barrier for bacterial invaders

The leaf environment has long been considered to be a hostile environment for bacteria. The leaf surface is exposed to rapidly fluctuating temperature and relative humidity, UV radiation, fluctuating availability of moisture in the form of rain or dew, lack of nutrients, and hydrophobicity (Lindow and Brandl, 2003). Such extreme fluctuations, for example within a single day, are certainly not experienced by pathogens in animal and human gut. Thus, it is tempting to speculate that animal pathogens may not even be able to survive and grow in an environment as dynamic as the leaf surface. However, the high incidence of human pathogens such as *S. enterica* and *E. coli* O157:H7 on fresh produce, sprouts, vegetables, leading to foodborne illness outbreaks indicate a certain level of human pathogen fitness in/on the leaf.

The plant surface presents a barrier to bacterial invaders by the presence of wax, cuticle, cell wall, trichomes, and stomata. All except stomata, present a passive defense system to prevent internalization of bacteria. Nonetheless, several bacteria are able to survive on and penetrate within the plant interior. The surface of just one leaf is a very large habitat for any bacteria. The architecture of the leaf by itself is not uniform and provides areas of different environmental conditions. There are bulges and troughs formed by veins, leaf hair or trichomes, stomata, and hydathodes that form microsites for bacterial survival with increased water and nutrient availability, as well as temperature and UV radiation protection (Leveau and Lindow, 2001; Miller et al., 2001; Brandl and Amundson, 2008; Kroupitski et al., 2009; Barak et al., 2011). Indeed, distinct microcolonies or aggregates of *S. enterica* were found on cilantro leaf surfaces in the vein region (Brandl and Mandrell, 2002) In addition, preference to the abaxial side of lettuce leaf by *S. enterica* may be is an important strategy for UV avoidance (Kroupitski et al., 2011). Conversion of cells to viable but non-culturable (VNBC) state in *E. coli* O157:H7 on lettuce leaves may also be a strategy to escape harsh environmental conditions (Dinu and Bach, 2011). Hence, localization to favorable microsites, avoidance of harsh environments, and survival by aggregation or conversion to non-culturable state may allow these human pathogens to survive and at times multiply to great extent on the leaf surface.

As stomata are abundant natural pores in the plant epidermis which serve as entrance points for bacteria to colonize the leaf interior (intercellular space, xylem, and phloem), several studies addressed the question as to whether human bacterial pathogens could internalize leaves through stomata. Populations of *E. coli* O157:H7 and *S. enterica* SL1344 in the Arabidopsis leaf apoplast can be as large as four logs per cm^2^ of leaf after surface-inoculation under 60% relative humidity (Roy et al., 2013) suggesting that these bacteria can and access the apoplast of intact leaves. Several microscopy studies indicated association of pathogens on or near guard cells. For instance, *S. enterica* serovar Typhimurium SL1344 was shown to internalize arugula and iceberg lettuce through stomata and bacterial cells were located in the sub-stomatal space (Golberg et al., 2011). However, no internalization of SL1344 was observed into parsley where most cells were found on the leaf surface even though stomata were partially open (Golberg et al., 2011). Cells of *S. enterica* serovar Typhimurium MAE110 (Gu et al., 2011), enteroaggregative *E. coli* (Berger et al., 2009b), and *E. coli* O157:H7 (Saldaña et al., 2011) were found to be associated with stomata in tomato, arugula leaves, and baby spinach leaves, respectively. In the stem *E. coli* O157:H7 and *Salmonella* serovar Typhimurium were found to be associated with the hypocotyl and the stem tissues including epidermis, cortex, vascular bundles, and pith when seedlings were germinated from contaminated seeds (Deering et al., 2011a,b).

The plant rhizosphere is also a complex habitat for microorganisms with different life styles including plant beneficial symbionts and human pathogens. Nutritionally rich root exudate has been documented to attract *S. enterica* to lettuce roots (Klerks et al., 2007a). Although bacteria cannot directly penetrate through root cells, sites at the lateral root emergence and root cracks provide ports of entry for *S. enterica* and *E. coli* O157:H7 into root tissues (Cooley et al., 2003; Dong et al., 2003; Klerks et al., 2007b; Tyler and Triplett, 2008), and in some instances between the epidermal cells (Klerks et al., 2007b). High colonization of *S. enterica* has been observed in the root-shoot transition area (Klerks et al., 2007b). Once internalized both bacterial pathogens have been found in the intercellular space of the root outer cortex of *Medicago truncatula* (Jayaraman et al., 2014). *Salmonella enterica* was found in the parenchyma, endodermis, pericycle, and vascular system of lettuce roots (Klerks et al., 2007b) and in the inner root cortex of barley (Kutter et al., 2006). A detailed study on the localization of *E. coli* O157:H7 in live root tissue demonstrated that this bacterium can colonize the plant cell wall, apoplast, and cytoplasm (Wright et al., 2013). Intracellular localization of *E. coli* O157:H7 seems to be a rare event as most of the microscopy-based studies show bacterial cells in the intercellular space only. Bacterial translocation from roots to the phyllosphere may be by migration on the plant surface in a flagellum-dependent manner (Cooley et al., 2003) or presumably through the vasculature (Itoh et al., 1998; Solomon et al., 2002). The mechanism for internal movement of enteric bacterial cells from the root cortex to the root vasculature through the endodermis and casparian strips and movement from the roots to the phyllosphere through the vascular system is yet to be demonstrated.

**Figure 1:**
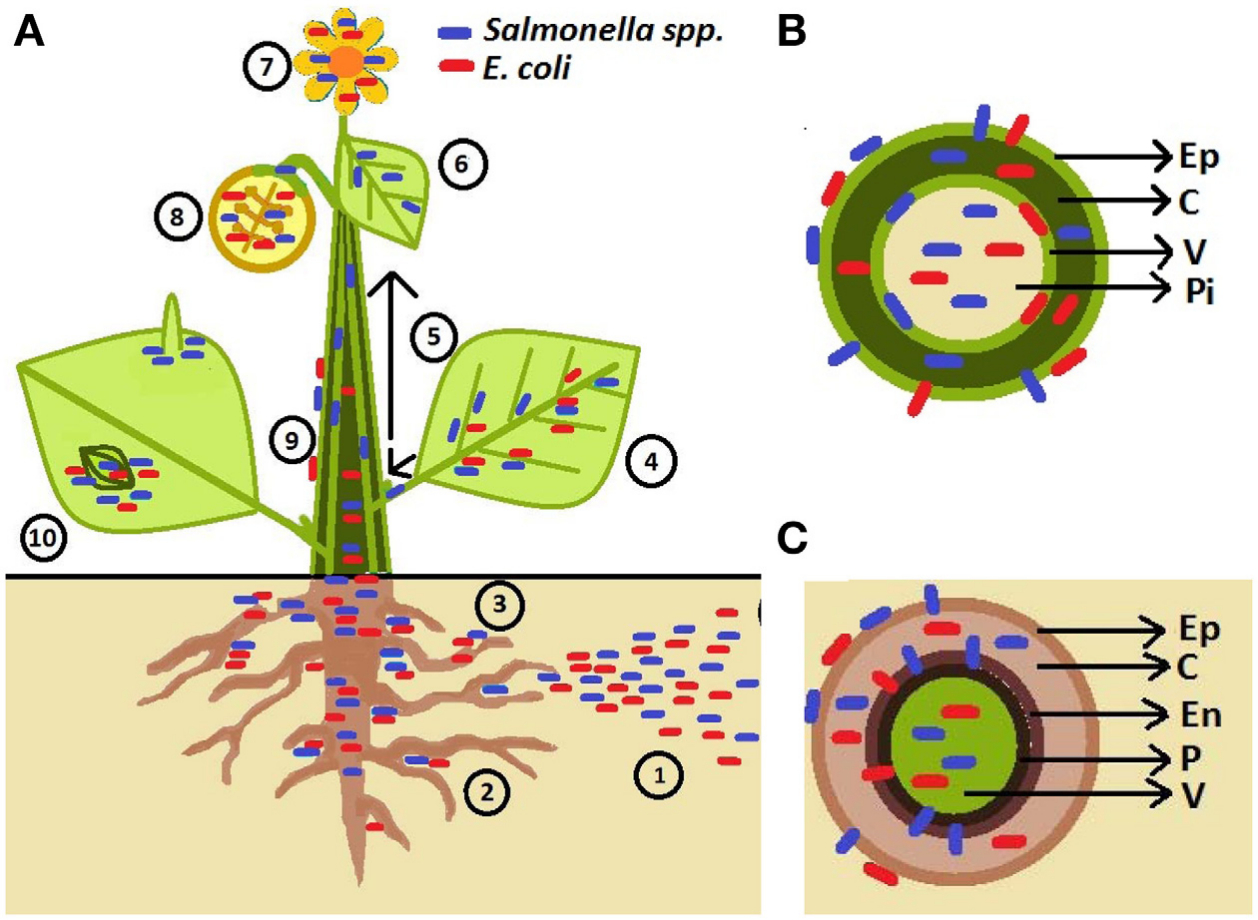
**Schematic representation of human pathogen (HP) association with plants**. **(A)** Pathogens are introduced to soil through contaminated irrigation water, fertilizers, manure, and pesticides (**1**). HPs are attracted to rhizosphere (**2**; Klerks et al., 2007a) and penetrate root tissues at the sites of lateral root emergence, root cracks as well as root-shoot transition area (**3**; Cooley et al., 2003; Dong et al., 2003; Klerks et al., 2007b; Tyler and Triplett, 2008). HPs were found to live on the leaf surface near veins (Brandl and Mandrell, 2002), in the leaf apoplast (intercellular space) (Brandl and Mandrell, 2002; Solomon et al., 2002; Niemira, 2007; Kroupitski et al., 2009; Barak et al., 2011; Dinu and Bach, 2011; Gu et al., 2011; Roy et al., 2013), and sometimes with affinity for abaxial side of leaf (e.g., *S. enterica*; (Kroupitski et al., 2011) (**4**). *Salmonella enterica* Typhimurium can enter tomato plants *via* leaves and move through vascular bundles (petioles and stems) (**5**) into non-inoculated leaves (**6**) and fruits (**8**) (Gu et al., 2011). HPs are also found to be associated with flower (**7**; Guo et al., 2001; Cooley et al., 2003). *Salmonella* could travel from infected leaves (**4**), stems (**5**), and flowers (**7**) to colonize the fruit interior (the diagram represents a cross-section of a fruit) and fruit calyx (**8**) Guo et al., 2001; Janes et al., 2005; Barak et al., 2011. *Escherichia coli* O157:H7 has also been observed in the internal parts of the apple and the seeds following contamination of the flower (**8**) (Burnett et al., 2000). Movement on the plant surface has also been observed (**9**; Cooley et al., 2003). Epiphytic *Salmonella* and *E. coli* O157:H7 can aggregate near stomata and sub-stomatal space (**10**; Shaw et al., 2008; Berger et al., 2009a,b; Golberg et al., 2011; Gu et al., 2011; Saldaña et al., 2011), reach the sub-stomatal cavity and survive/colonize in the spongy mesophyll (Solomon et al., 2002; Wachtel et al., 2002; Warriner et al., 2003; Jablasone et al., 2005; Franz et al., 2007). *Salmonella* cells were observed near trichomes (**10**; Barak et al., 2011; Gu et al., 2011). **(B)** Stem cross-section showing bacteria located in different tissues (Ep, epidermis; C, cortex; V, vascular tissue; Pi, pith) (Deering et al., 2011a,b). **(C)** Root cross-section showing bacteria on the root surface, internalizing between the epidermal cells, and colonizing root outer and inner cortex, endodermis (En), pericycle (P) and vascular system (Kutter et al., 2006; Klerks et al., 2007a,b; Jayaraman et al., 2014).

Several outbreaks of *S. enterica* have also been associated with fruits, especially tomatoes. *Salmonella enterica* is unlikely to survive on surface of intact fruits (Wei et al., 1995) raising the question: what are the routes for human pathogenic bacteria penetration into fruits? It has been suggested that *S. enterica* can move from inoculated leaves (Barak et al., 2011), stems, and flowers (Guo et al., 2001) to tomato fruits. However, the rate of internal contamination of fruits was low (1.8%) when leaves were surface-infected with *S. enterica* (Gu et al., 2011). The phloem has been suggested as the route of movement of bacteria to non-inoculated parts of the plant as bacterial cells were detected in this tissue by microscopy (Gu et al., 2011). Figure 1 depicts the observed phyllosphere and rhizosphere niches colonized by bacteria in/on intact plants and probable sources of contamination.

## Perception of human pathogens by the plant immune system

Plants possess a complex innate immune system to ward off microbial invaders (Jones and Dangl, 2006). Plants are able to mount a generalized step-one response that is triggered by modified/degraded plant products or conserved pathogen molecules. These molecules are known as damage or pathogen associated molecular patterns (DAMP/PAMP). In many cases, conserved PAMPs are components of cell walls and surface structures such as flagellin, lipopolysaccharides, and chitin (Zeng et al., 2010). Examples of intracellular PAMPs exist such as the elongation factor EF-Tu (Kunze et al., 2004). PAMPs are recognized by a diverse set of plant extracellular receptors called pattern-recognition receptors (PRRs) that pass intracellular signals launching an army of defense molecules to stop the invasion of the pathogens. This branch of the immune system known as pathogen-triggered immunity (PTI) is the first line of active defense against infection.

Human pathogen on plants (HPOP) is an emerging field that only recently has caught the attention of plant biologists and phytopathologists. A few studies have been reported in the last 5–10 years, which focused on the most well studied PAMPs, flagellin and lipopolysaccharide (LPS), in the interaction of human pathogens with plants. Table 1 lists the plants, bacterial strains, and method details for such studies.

**Table 1:** **Experimental conditions used in the studies reporting plant response to pathogenic *Salmonella* and *E. coli***.

**References**	**Plant**	**Plant genotype**	**Wild type bacterium**	**Mutant bacterium**	**Plant tissue used for detection**	**Inoculation method**	**Inoculum concentration**	**Surface sterilization**	**Methods**	**Intact plant tissue for infection**
**FLAGELLIN AND LPS PERCEPTION**										
Cooley et al., 2003	*A. thaliana*	Col-0	*S. enterica* serovar Newport (RM1655)*; E. coli* O157:H7 Odwalla (RM1484); *Enterobacter asburiae* (RM3638)	*ΔflhDC, flaN*	Roots and shoots of seedling and adult plants, leaves, flowers, seeds, chaff	Soil, seed, root inoculation	1 × 10^4^ or 1 × 10^6^ cfu/ml for root inoculation; 1 × 10^8^ cfu/ml for seed inoculation and 1 × 10^8^ cfu/g for soil inoculation	No	Microscopy, plating	Yes
^*^Iniguez et al., 2005	*A. thaliana, M. truncatula*, wheat*, M. sativa*	Wheat Trenton; *M. truncatula* Jester, Jermalong, Gaerten A17, Mutant Sickle; *M. sativa* CUS101; *A. thaliana* Col-0, *npr1-4, nahG*	*S. enterica* serovar Typhimurium 14028s	*spaS, sipB, fliC, fljB*	Root and hypocotyl	Seedling inoculation	dose response	Yes	Plating	Yes
^*^Shirron and Yaron, 2011	Tobacco		*S. enterica* serovar Typhimurium SL1344, 14028s	*ΔinvA, ΔrfaH, ΔphoP*	Leaf	Syringe infiltration; drip-irrigation	7.5 log cfu/ml	No	Microscopy	Yes
^*^Seo and Matthews, 2012	*A. thaliana*	Col-0, *npr1-1*	*E. coli* O157:H7 43895, 86-24	*ΔfliC, ΔcsgD*, Δ*waaI*	Whole plant	Dipping	1 × 10^8^ cfu/ml	No	Plating	Yes
^*^Garcia et al., 2014	*A. thaliana*	Col-0, *fls2*	*S. enterica* serovar Typhimurium 14028s, SL1344; *S. enterica* serovar Senftenberg 20070885	*prgH, fliC, fljB, hrcC*	Seedling	Seedling inoculation	2 × 10^8^ cfu/ml	No	Plating	Yes
Meng et al., 2013	Tobacco, tomato		*S. enterica* serovar Typhimurium 14028s	*ΔfliC, ΔfljB, ΔhilA, Δ* hilD, *ΔsirA, ΔssrAB, ΔsseA, ΔsseB, ΔinvA, ΔprgH*	Leaf	Syringe infiltration	2 × 10^4^ cfu/ml	No	Plating	Yes
**STOMATAL IMMUNITY**										
Melotto et al., 2006	*A. thaliana*	Col-0, *fls2*	*E. coli* O157:H7		Leaf	Epidermal peels	1 × 10^8^ cfu/ml		Microscopy	No
Kroupitski et al., 2009	Lettuce	Iceberg	*S. enterica* serovar Typhimurium SL1344	*fliGHI, cheY*	Leaf	Leaf pieces submersion	1 × 10^8^ cfu/ml	No	Microscopy	No
^*^Roy et al., 2013	*A. thaliana*, lettuce	*A. thaliana* Col-0 *ost1-2*, Butter Lettuce	*S. enterica* serovar Typhimurium SL1344; *E. coli* O157:H7		Leaf	Dipping	1 × 10^8^ cfu/ml	Yes	Microscopy, plating	Yes
**PLANT INTRACELLULAR RESPONSE**										
Thilmony et al., 2006	*A. thaliana*	Col-0	*E. coli* O157:H7	*fliC*	Leaf	Vacuum infiltration	1 × 10^8^ cfu/ml			
Klerks et al., 2007b	Lettuce	Tamburo	*S. enterica* serovars Dublin; *E. coli* JM109		Seedling phyllosphere	Manure contaminationSeedling inoculation	1 × 10^7^ cfu/g manure or 1 × 10^7^ cfu/ml inoculum	Yes	Plating	Yes
Schikora et al., 2008	*A. thaliana*	Col-0	*S. enterica* serovar Typhimurium 14028s		Seedling, leaf	Seedling inoculation, leaf vacuum infiltration	3 × 10^8^ cfu/ml	Yes	Plating	Yes
Saldaña et al., 2011	Baby spinach		*E. coli* O157:H7	*escN, tir, eae, espFu, espP, fliC, qseB, hcpA, ecpA, elfA, csgA, csgD, bscA*	Leaf	Leaf pieces submersion	1 × 10^7^ cells	Yes for some experiments	Microscopy, plating	No
Schikora et al., 2011	*A. thaliana*	Col-0	*S. enterica* serovar Typhimurium 14028s	*prgH, invA, ssaV, ssaF*	Leaf	Syringe infiltration	1.7 × 10^8^ cfu/ml	Yes	Plating	Yes
Üstün et al., 2012	Tomato, tobacco, pepper	Tomato Money maker; tobacco Domin., pepper ECW-10R	Transient expression of *S. enterica* SseF in tobacco		Leaf	Transient expression by syringe infiltration of vector organism	2 × 10^8^ cfu/ml		Plating	Yes
Jayaraman et al., 2014	*M. truncatula*		*S. enterica*. serovars Schwarzengrund, Enteritidis, Mbandaka, Havana, Cubana; *E. coli* O157:H7 serovars Odwalla, EDL933, H2439, C7927, 96A 13466		Root	seedling root	Dose response	Yes for some experiments	Microscopy, plating	Yes
**GENOTYPIC VARIABILITY**										
Barak et al., 2008	Radish, tomato, broccoli, turnip, carrot, lettuce, cilantro, parsley, spinach, radicchio, endive	Lettuce Balady Aswan, Salinas 88, Little Gem, PI251246, Pavane, Valmaine, Iceburg, La Brillante, Paris Island, Parade, Calmar; Tomato Brandywine, Amish Paste, Money Maker, Rose, Soldacki, Stupics, Green Grape, San Marzano, Nyarous, Yellow Pear	*S. enterica* serovars Baildon 05x-02123, Cubana 98A9878, Enteritidis 99A-23, Havana 98A4399, Mbandaka 99A1670, Newport 96E01152C-TX, Poona 00A3563, Schwarzengrund 96E01152C		Seedling phyllosphere	Soil inoculation	1 × 10^4^ cfu/ml	No	Plating	Yes
Brandl and Amundson, 2008	Lettuce	Parris Island	*E. coli* O157:H7 H1827; *S. enterica* serovar Thompson RM1987		Leaf	Dip inoculation	1 × 10^5^ cfu/ml	No	Microscopy, plating	Yes
Mitra et al., 2009	Spinach	Bordeaux, Tyee, Space	*E. coli* O157:H7		Leaf, stem, root	Leaf drop, leaf infiltration, soil drench, stem puncture	1 × 10^6^ cfu/ml	Yes	Microscopy, plating, BAX PCR assay	Yes
Barak et al., 2011	Tomato	H7996, Yellow Pear, Nyagous, LA2838A, LA3172, LA3556, LA0337, LA1049, Micro-Tom, Money Maker	*S. enterica* serovars Baildon 99A 23, Cubana 98A 9878, Enteritidis 05x-02123, Havana 98A4399, Mbandaka 99A 1670, Newport 96 E01153c-TX, Poona 00A 3563, Schwarzenfrund 96 E01152c-TX		Seedling phyllosphere and leaf	Soil inoculation; water inoculation; leaf dipping	1 × 10^8^ cfu/ml	No	Microscopy, plating, BAX PCR assay	Yes
Golberg et al., 2011	Lettuce, arugula, parsley, tomato, basil	Iceberg, Romaine, Red Ruby	*S. enterica* serovar Typhimurium SL1344		Leaf	Leaf pieces/leaf submersion	1 × 10^8^ cfu/ml	No	Microscopy	Leaf pieces and intact leaves

* *Indicates articles that have also reported plant intracellular responses to bacteria*.

### Flagellin perception

Flagellin, the structural component of flagellum in bacteria, is involved in bacterial attachment and motility on the plant (Cooley et al., 2003), is recognized by plant through the FLS2 receptor (Garcia et al., 2014), and induces plant defenses (Meng et al., 2013; Garcia et al., 2014). Similar to the well-studied PTI elicitor flg22 (Felix et al., 1999), the flg22 epitope of *S. enterica* serovar Typhimurium 14028 is also an effective PAMP and elicitor of downstream immune responses in Arabidopsis (Garcia et al., 2014), tobacco, and tomato plants (Meng et al., 2013). Flagellum-deficient mutants of *S. enterica* serovar Typhimurium 14028 are better colonizers of wheat, alfalfa, and Arabidopsis roots as compared to the wild type bacterium (Iniguez et al., 2005) further suggesting that the *Salmonella* flagellum induces plant defenses that may restrict bacterial colonization of several plant organs. However, the *Salmonella* flg22 peptide is not the only PAMP for elicitation of plant immune response as *fls2* mutant of Arabidopsis still shows a low level of PTI activation in response to this PAMP (Garcia et al., 2014).

Purified flagellin or derived epitopes of *E. coli* O157:H7 has not been used to induce plant defenses. However, flagellum-deficient mutant of this strain does not activate the SA-dependent *BGL2* gene promoter as much as the wild type strain and shows larger population in Arabidopsis than the wild type strain (Seo and Matthews, 2012) further suggesting that surface structures in the bacterial cell are perceived by plants.

The differences in responses observed could be attributed to the presence of other microbial signatures eliciting plant defense. Variations in plant response to *S. enterica* flagellin could be owed to host-strain specificity as well. Although flagellin sequences from *S. enterica* strains and other bacteria are highly conserved, even a minor change of five amino acids in the flg22 epitope leads to reduced activation of PTI in Arabidopsis, tobacco, and tomato plants (Garcia et al., 2014). Adding to the specificity, it has also been shown that Brassicaceae and Solanocecae plants recognize specific flagellin (Robatzek et al., 2007; Clarke et al., 2013). Hence, evolving variations in flagellin sequences could be a strategy employed by the pathogens to avoid plant recognition, which in turn leads to the development of pathogen-specific immune responses in the plant.

Flagella also play an important role in bacterial behavior on the plant. Several studies have pointed out to the usefulness of flagella for attachment to leaf surfaces and movement on plant surfaces (Berger et al., 2009a,b; Xicohtencatl-Cortes et al., 2009; Saldaña et al., 2011; Shaw et al., 2011).

### LPS perception

Lipopolysaccharide (LPS) is a component of the cell wall of Gram-negative bacterial pathogens of animals and plants. In the animal host, LPS is a well-characterized PAMP that is recognized by host Toll-like receptor 4 (de Jong et al., 2012). In plants however, receptors for LPS have not been discovered yet. Nonetheless, current evidence suggests that human pathogen-derived LPS can be perceived by plants resulting in PTI activation. For instance, on the leaf surface, purified LPS from *Pseudomonas aeruginosa, S. Minnesota* R595, and *E. coli* O55:B5 induces strong stomatal closure in Arabidopsis (Melotto et al., 2006). Purified LPS from *Salmonella* triggers of ROS production and extracellular alkalinization in tobacco cell suspension (Shirron and Yaron, 2011) but not on tomato leaves (Meng et al., 2013) suggesting that LPS recognition may be either dependent on experimental conditions or variable among plant species.

Genetic evidence suggests that the high activity of SA-dependent *BGL2* gene promoter in Arabidopsis is dependent on the presence of LPS in *E. coli* O157:H7 as higher activity of this promoter was observed in the wild type bacterial as compared to its LPS mutant (Seo and Matthews, 2012). However, LPS-dependent responses seem not to be sufficient to restrict bacterial survival on plants as the population titer of *E. coli* O157:H7 LPS mutant or wild type in plant is essentially the same (Seo and Matthews, 2012). Additionally, live *S*. Typhimurium cells do not induce ROS in epidermal tissue of tobacco (Shirron and Yaron, 2011) suggesting that, at least *Salmonella*, can suppress LPS-induced ROS and extracellular alkalinization.

Similar to flagellin, the O-antigen moiety of LPS is not only important for plant perception of bacterial cells, but also for bacterial attachment, fitness, and survival on plants (Barak et al., 2007; Berger et al., 2011; Marvasi et al., 2013).

### Functional output of bacterium perception

One of the earliest PTI responses in plants is stomatal closure that greatly decreases the rate of pathogen entry into plant's internal tissues. This response requires molecular components of PTI including such as flagellin and LPS perception and hormone perception and signaling (Melotto et al., 2006, 2008; Zeng and He, 2010; Sawinski et al., 2013). Stomatal immunity is also triggered by the presence of human pathogens *S. enterica* serovar Typhimurium SL1344 and *E. coli* O157:H7 (Melotto et al., 2006; Kroupitski et al., 2009; Roy et al., 2013), albeit at various levels. For instance, *E. coli* O157:H7 induces a strong stomatal immunity and *Salmonella* SL1344 elicits only a transient stomatal closure in both Arabidopsis (Melotto et al., 2006; Roy et al., 2013) and lettuce (Kroupitski et al., 2009; Roy et al., 2013) suggesting that the bacterial strain SL1344 can either induce weaker or subvert stomata-based defense. Active suppression of stomatal closure by SL1344 may be unlikely because it cannot re-open dark-closed stomata (Roy et al., 2013). However, it is possible that signaling pathways underlying bacterium-triggered and dark-induced stomatal closure are not entirely overlapping and SL1344 acts on immunity-specific signaling to subvert stomatal closure.

## Plant intracellular response to human pathogens

Recognition of PAMPs by PRRs leads to several hallmark cellular defense responses that are categorized based on the timing of response. Zipfel and Robatzek (2010) have discussed that early responses occur within seconds to minutes of recognition including ion fluxes, extracellular alkalinization, and oxidative burst. Intermediate responses occur within minutes to hours including stomatal closure, ethylene production, mitogen-activated protein kinase (MAPK) signaling, and transcriptional reprogramming. Late responses occur from hours to days and involve callose deposition, salicylic acid accumulation, and defense gene transcription.

**Figure 2:**
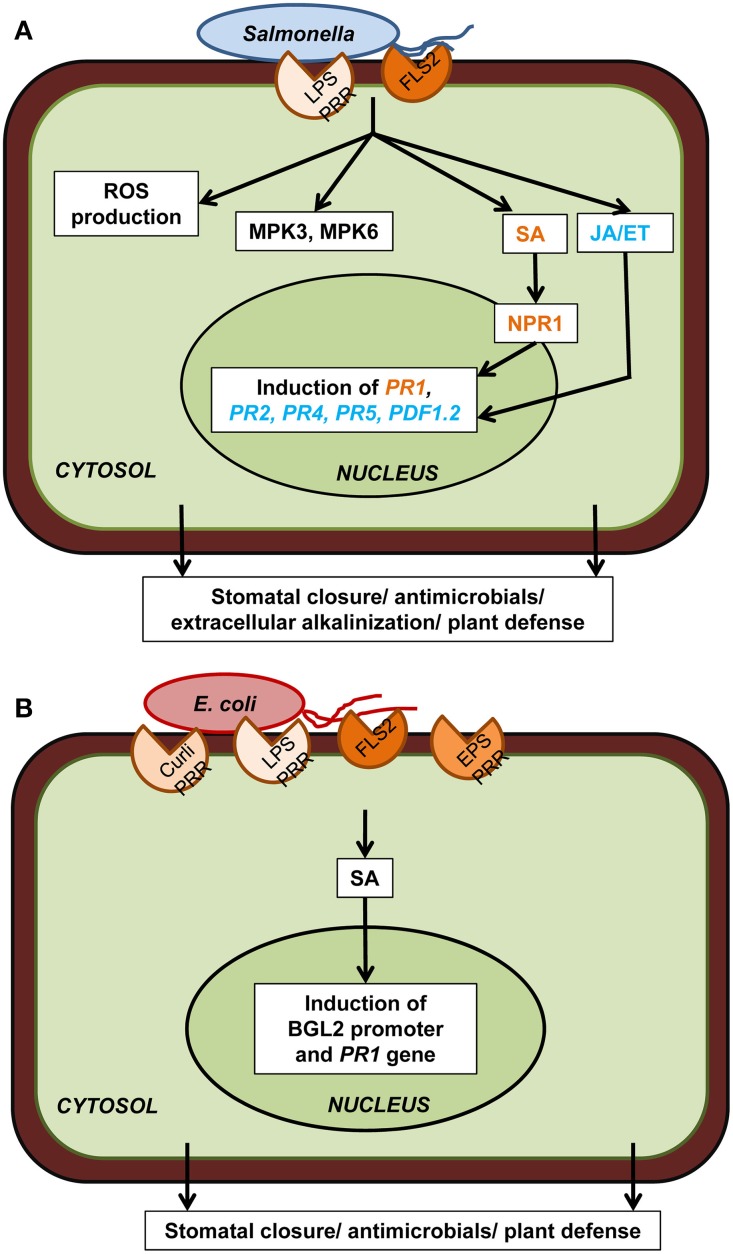
**Plant cellular defense responses against human pathogens. (A)** Upon reception of PAMP (flagellin, LPS) through PRR (FLS2 and putatively others), *Salmonella* spp. trigger downstream plant defense responses which include ROS production, MPK3/6, salicylic acid (SA) signaling through NPR1, jasmonic acid (JA) and ethylene (ET) signaling, defense-associated gene induction, and extracellular alkalinization. All these cellular events ultimately lead to stomatal closure, antimicrobial activity, and plant defense. **(B)** *Escherichia coli* PAMPs (curli, LPS, flagellin, EPS) are also perceived by PRRs (FLS2 and putatively others) present on plant cell surface which triggers the induction of the SA-dependent BGL2 promoter activity and *PR1* gene expression. Only components that have been directly demonstrated experimentally are included in the diagram. Plant defense responses in case of both these human pathogens are strain specific as well as plant cultivar specific.

These hallmark plant cellular defenses have also been tested for both *E. coli* and *S. enterica* (Figure 2). In particular, *S. enterica* infection results in the induction of *MPK3*/*MPK6* kinase activity and plant defense-associated genes *PDF1.2, PR1*, and *PR2* in Arabidopsis leaves (Schikora et al., 2008) as well as *PR1, PR4*, and *PR5* in lettuce (Klerks et al., 2007b). MPK6 activation in Arabidopsis is independent of FLS2 (Schikora et al., 2008), indicating that flagellin is not the only active PAMP of *Salmonella* and plant response to other PAMPs may converge at MAPK signaling. Direct comparison of the *PR1* gene expression in Arabidopsis indicated that both *E. coli* O157:H7 and *Salmonella* SL1344 are able to induce this defense marker gene, however at difference levels (Roy et al., 2013). The *PR1* gene induction is low in SL1344-infected plants indicating that immune responses are either weaker or are suppressed by *Salmonella*.

A few studies (Table 1) have addressed the role of plant hormones in response to endophytic colonization of human bacterial pathogens:

### Ethylene signaling

The ethylene-insensitive mutant of Arabidopsis, *ein2*, supports higher *Salmonella* 14028 inside whole seedlings as compared to the wild type Col-0 plants (Schikora et al., 2008). Furthermore, addition of a specific inhibitor of ethylene mediated signaling, 1-methylcyclopropene (1-MCP), to the growth medium resulted in increased *S*. *enterica* 14028 endophytic colonization of *Medicago truncatula*, but not *M. sativum*, roots and hypocotyls (Iniguez et al., 2005) suggesting that the role of endogenous ethylene signaling maybe be specific to each plant-bacterium interaction. However, ethylene signaling may play a contrasting role during fruit contamination. Tomato mutants (*rin* and *nor*) with defects in ethylene synthesis, perception, and signal transduction show significantly reduced *Salmonella* proliferation within their fruits as compared to the wild type control (Marvasi et al., 2014).

### Jasmonic acid

Similar to the *ein2* mutant, the coronatine-insensitive mutant of Arabidopsis, *coi1-16*, also supports high *Salmonella* 14028 inside whole seedlings (Schikora et al., 2008). Along with the induction of the jasmonate-responsive gene *PDF1.2* addressed in the same study and mentioned above, it seems that jasmonate signaling is also an important component to restrict *Salmonella* infection in, at least, Arabidopsis. These results are surprising as *coi1* mutants are well known to have increased resistant to various bacterial pathogen of plants, such as *P. syringae*, but not to fungal or viral pathogens (Feys et al., 1994; Kloek et al., 2001).

### Salicylic acid

Two genetic lines of Arabidopsis has been extensively used to determine the role of salicylic acid (SA) in plant defenses against phytopathogens, the transgenic *nahG* plant that cannot accumulate SA (Friedrich et al., 1995) and the null mutant *npr1* that is disrupted in both SA-dependent and -independent defense responses (Ton et al., 2002). Both of these plant lines support higher populations of *Salmonella* 14028 inside their roots (Iniguez et al., 2005) and seedlings (Schikora et al., 2008) as compared to the wild type plant. NPR1-dependent signaling is important reduce the population of the curli-negative strain of *E. coli* O157:H7 43895 but not for the curli-positive strain 86-24 in Arabidopsis leaves (Seo and Matthews, 2012). Although only a few strains of *Salmonella* and *E. coli* have been used, there is an emerging patterns suggesting that SA itself and activation of SA-signaling can potentially restrict HPOP.

In attempts to understand the overall cellular transcriptional response to human bacterial pathogens, global transcriptomic analyses have been used. Thilmony et al. (2006) showed that *E. coli* O157:H7 regulates PTI-associated genes in Arabidopsis leaves, albeit in a flagellin-independent manner. A similar transcriptomic analysis with medium-grown Arabidopsis seedlings 2h after inoculation with *S. enterica* serovar Typhimurium 14028, *E. coli* K-12, and *P. syringae* pv. *tomato* DC3000 showed a strong overlap among genes responsive to each bacterial infection suggesting a common mechanism of plant basal response toward bacteria (Schikora et al., 2011). Gene expression analysis of *Medicago truncatula* seedlings root-inoculated with only two bacterial cells per plant indicated that 83 gene probes (30–40% of each data set) were commonly regulated in response to *S. enterica* and *E. coli* O157:H7 (Jayaraman et al., 2014). All together, these studies indicate that each human pathogenic bacterium can modulate specific plant genes beyond a basal defense response; however the mechanisms for plant-bacterium specificity are largely unknown.

## Can human pathogenic bacteria induce ETI in plant cells?

Successful virulent pathogens of plants are able to defeat this army plant defense by employing its own set of artillery (such as the type three secretion system effectors and phytotoxins) and cause disease in the host plant (Melotto and Kunkel, 2013; Xin and He, 2013). In incompatible interactions (i.e., low bacterial colonization and no disease on leaves), the host plant already has pre-evolved molecules (R proteins) that recognize these effectors and cause a specific defense response to this pathogen. This specific response is called effector-triggered immunity (ETI). Because the type 3-secretion system (T3SS) is important for the virulence of both animal and plant pathogenic bacteria on their natural hosts as evidenced by the use of bacterial mutants, it is reasonable to expect that T3SS would be important for HPOP as well. However, animal and plant cell surfaces are structurally different; the plant cells wall seems to be impenetrable by the secretion needle of the extracellular animal pathogens (*Salmonella* and *E. coli*) as discussed by He et al. (2004) raising the question of how these effectors can reach the plant cytoplasm and interfere with plant defenses. To date, there is no evidence for the ability of human pathogens to inject T3SS effectors inside plant cells. It is possible that the T3SS is still active on the plant cell surface and the effectors are secreted into the plant apoplast. If that is the case, however, plant membrane receptors would be necessary to recognize the effectors and trigger plant cellular responses. Nevertheless, it has been observed that the T3SS mutant of *E. coli* O157:H7, *escN*, has reduced ability to attach to and colonize baby spinach leaves similar to the *fliC* mutant (Saldaña et al., 2011). Furthermore, apoplastic population of T3SS structural mutants of *S. enterica* serovar Typhimurium 14028 (*invA, prgH, ssaV*, and *ssaJ*) is smaller than that of the wild type bacterium in Arabidopsis leaves (Schikora et al., 2011) and plant defense-associated genes are up-regulated for longer time by the *prgH* mutant than wild type *Salmonella* in Arabidopsis seedlings (Garcia et al., 2014). Contrary to these findings, Iniguez et al. (2005) reported that two *Salmonella* 14028 T3SS-SPI1, the structural mutant *spaS* and the effector mutant *sipB*, hypercolonize roots and hypocotyls of *M. sativum* and fail to induce SA-dependent *PR1* promoter in Arabidopsis leaves. More studies need to be conducted to conclude whether T3SS of *Salmonella* acts as “recognizable” surface structure similar to flagellum and/or as a conduit to deliver effectors in plant tissues and trigger ETI. It is worth mentioning that T3SS and effectors of the phytopathogen *P. syringae* pv. *syringae* have functions on ETI as well as bacterial fitness on plant surface (Lee et al., 2012) and the filamentous T3SS protein EspA is required for *E. coli* O157:H7 attachment to arugula leaves (Shaw et al., 2008).

The *invA* structural mutant, that is defective in all T3SS-1 system-associated phenotypes, induces high ROS and extracellular alkalinizing in tobacco BY-2 cell suspension and hypersensitive reaction (HR) in tobacco leaves as compared to the wild type strain (Shirron and Yaron, 2011) suggesting that T3SS is important for this suppression of immunity. However, Shirron and Yaron (2011) also reported that plant response to the regulatory mutant *phoP* that modulates the expression of many effector proteins and membrane components (Dalebroux and Miller, 2014), is no different to that of the wild type bacterium. These findings raised the question whether the phenotypes observed are due to the T3SS structure itself or due to the translocated effectors. A recent report shows that transient expression of the type three effector of *Salmonella* 14028 SseF in tobacco plants elicits HR, and this response is dependent on the SGT1 protein (Üstün et al., 2012). This study suggests that SseF can induce resistant-like response in plants and requires resistance (R) protein signaling components. Üstün et al. (2012) and Shirron and Yaron (2011) also showed that *Salmonella* 14028, which is able to deliver the SseF effector, cannot induce HR or any disease-like symptoms in tobacco leaves. Thus, it remains to be determined what would be the biological relevance of ETI in the *Salmonella* and other human pathogenic bacteria in their interaction with plants in nature.

## Genotypic variability in plant-*salmonella* and plant-*E. coli* interactions

Although *S. enterica* and *E. coli* O157:H7 have not been traditionally known to be closely associated with plants and modulate plant's physiology, the evidence tells us otherwise. An arms-race evolution in both the human pathogen and the plant is therefore, expected. A few studies (methodology details described in Table 1) have addressed whether genetic variability among plant species or within the same plant species (i.e., cultivars, varieties, and ecotypes) can be correlated with differential bacterial behavior and/or colonization of plants. Barak et al. (2011) described that different tomato cultivars can harbor different levels of *S. enterica* population after inoculation *via* water (sprinkler imitation) indicating plant factors may control the ability of bacterial to colonize the phyllosphere. However, they also found that the cultivar with the smallest *S. enterica* population also had the lowest number of speck lesions when infected with the tomato pathogen *Pst* DC3000 (Barak et al., 2011), suggesting that strong basal defense in this cultivar may account for low bacterial colonization. On a comparative study of *S. enterica* contamination of several crop species, Barak et al. (2008) reported that seedlings from Brassicaceae family have higher contamination than carrot, tomato, and lettuce when grown on contaminated soil. Seedling contamination correlated with the *Salmonella* population in the phyllosphere of all crop species, except tomato.

Golberg et al. (2011) reported variations in internalization of *Salmonella* SL1344 in different leafy vegetables and fresh herbs using confocal microscopy. Internalization incidence (% of microscopic fields containing bacterial cells) was high in iceberg lettuce and arugula, moderate in romaine lettuce, red lettuce, basil, and low in parsley and tomato. Attraction to stomata was seen in iceberg lettuce and basil, not in arugula, parsley, and tomato. Brandl and Amundson (2008) reported that the age of romaine lettuce leaves is correlated with population size of *E. coli* O157:H7 and *S. enterica* Thompson on leaves. Young leaves (inner) harbor greater number of cells than middle aged leaves. These authors also observed that exudates on the surface of younger leaves have higher nitrogen content than that of older leaves, which may contribute to determining the bacterial population size on the leaf. Thus, it is tempting to speculate that the genetic variability existent among plant genotypes regarding the chemical composition of their organ exudates may be a determinant for human pathogen behavior (such as chemotaxis and tropism toward stomata and roots) and ability to colonize plants.

Finally, Mitra et al. (2009) studied the effect of different methods of inoculation on internalization and survival of *E. coli* O157:H7 in three cultivars of spinach. Among the organs studied, the spinach phylloplane and the stem provided the most and least suitable niche for this bacterium colonization, respectively. Although the leaf surface was the best “territory” for *E. coli*, the leaf morphologies of each cultivar affected the ability of this bacterium to survive.

Collectively, all these studies point out that the plant genotype, age, leaf morphology, chemical composition of exudates, and the primarily infected organ affect the outcome of bacterial colonization of plants and the process may not be a generalized phenomenon, consequently shaping specific human pathogen and plant interactions.

## Concluding remarks

The fundamental understanding of plant association with human bacterial pathogens that do not cause visual or macroscopic symptom in the plant, but yet are major food contaminants, are in its infancy. Both plant and bacterial factors are critical for these cross-kingdom interactions and emerging evidence suggests an overlap between plant molecular responses to human pathogens and phytopathogens. The future challenge will be to determine how these interactions differ. As this field of research is relatively new, we see differences in conclusions from different laboratories regarding multiplication vs. decline in bacterial populations overtime and disease-like symptoms vs. HR on inoculated plants. These differences are mainly associated with differences in methods of inoculation, bacterial strains, inoculum concentration, plant age, and plant cultivation methods (e.g., growth on medium, soil, or hydroponic solutions). Standard procedures for model systems, consensus, and collaborations must be developed among food scientists, microbiologists, plant pathologists, and molecular biologists to elucidate the specificity of each plant-bacterium interaction and avoid discrepancies in making general conclusions. A major point to be resolved is whether the observed plant defenses against *Salmonella* and its PAMPs are due to low recognition and/or active suppression. If *Salmonella* suppression of the plant immunity is a cause of weak defense responses, the major question becomes what is the responsible factor? This line of research might lead to a whole new paradigm that otherwise could not be revealed by only studying plant associations with its own natural pathogens.

### Conflict of interest statement

The authors declare that the research was conducted in the absence of any commercial or financial relationships that could be construed as a potential conflict of interest.
